# From Lineage to Longevity: A Field Guide to the Key Players in Epigenetic Contribution to Offspring Health

**DOI:** 10.3390/cimb47050323

**Published:** 2025-04-30

**Authors:** Razia Zakarya

**Affiliations:** 1 School of Life Sciences, University of Technology Sydney, Sydney 2007, Australia; razia.zakarya@uts.edu.au; 2 Epigenetics of Chronic Disease Group, Woolcock Institute of Medical Research, Macquarie University, Sydney 2113, Australia

**Keywords:** DNA methylation, histone modification, development, epigenetics

## Abstract

Epidemiological evidence firmly supports the rationale that chronic diseases demonstrate a heritability component. Notwithstanding recent advances in genomic technologies, in a significant proportion of heritable diseases, a candidate gene of interest that explains the entire picture of heritability remains to be identified. Further epidemiological evidence points to environmental risk factors contributing to chronic disease prevalence and severity. The Developmental Origins of Health and Disease hypothesis points to epigenetics as the mechanism modulating gene–environment interactions to elicit disease. Yet the primary effector of epigenetic inheritance remains to be elucidated. This review focuses on key contributors to mammalian development and the epigenetic changes measured therein, to draw attention towards potential molecular candidates underpinning chronic disease heritability.

## 1. Introduction

Chronic noncommunicable diseases are the leading health concern of the modern age, with three out of four deaths in 2019 [1] caused by them, and millions of people living with a chronic disease. This prevalence poses a significant healthcare and loss of productivity cost that is estimated to grow as large as USD 47 trillion by 2030 [2]. Common chronic diseases include those that affect the cardiovascular, respiratory, endocrine, digestive, and hepatic systems, with cardiovascular diseases, diabetes mellitus, and respiratory diseases causing the highest number of deaths [3].

Interestingly, chronic noncommunicable diseases share common risk factors, which may be metabolic, modifiable behavioural, or environmental. Examples of each include hypertension, tobacco use, and air pollution, respectively [4]. There is a hereditary link in chronic diseases, but genetic susceptibility loci only explain a small proportion of prevalence and are also present in healthy populations. One such example is in asthma, a chronic health condition wherein heritability is estimated to range from 40 to 90% [5,6,7,8,9], yet susceptibility loci [5] are present in healthy populations and do not account for the majority of prevalence patterns [6,7,8]. As such, it is postulated that chronic diseases are driven by complex gene–environment interactions [9,10] which remain to be understood.

The Developmental Origins of Health and Disease (DOHaD) hypothesis proposes that the leading cause of pathological gene–environment interactions are epigenetic changes induced by environmental insults during early life development [11,12,13,14]. Furthermore, an increasing body of evidence shows that a range of stressors occurring during critical stages of embryo development can adversely impact the fidelity of epigenetic reprogramming [15,16] and may serve as the basis for the homeostatic instability we recognise as chronic diseases, such as cardiovascular disease [17,18], diabetes [19], and asthma [20,21]. This review will draw together key points on epigenetic mechanisms, the role of epigenetics in development, and epigenetic heritability. In doing so, it will point to potential epigenetic mechanisms of chronic disease susceptibility caused by the developmental environment.

## 2. Epigenetic Mechanisms

**Figure 1:**
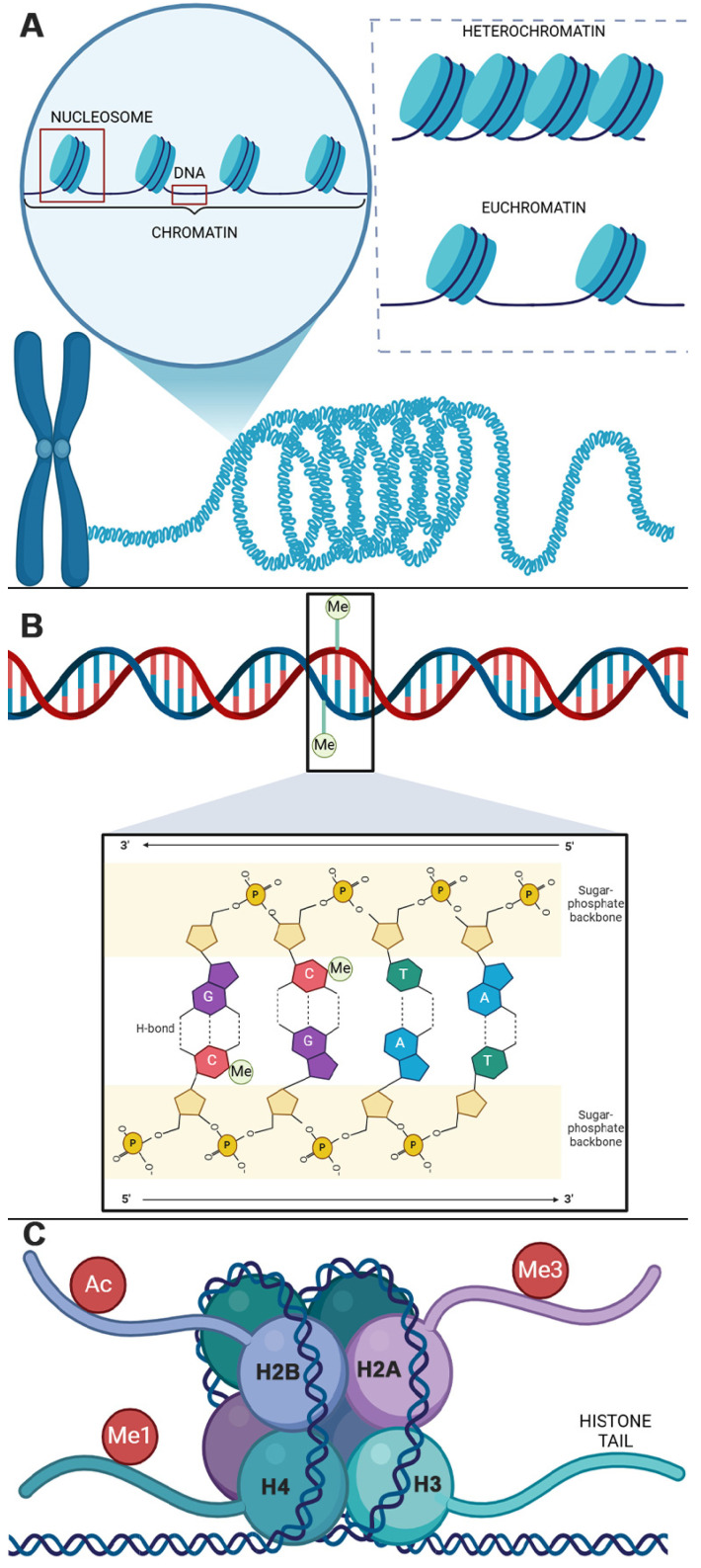
Schematic representation of epigenetic concepts. (**A**) Chromosomal DNA unwound to reveal chromatin, comprising DNA (black strand) wrapped around histone (teal disc) to form nucleosome units. Compacted nucleosome units result in tightly wound chromatin (heterochromatin) and spaced nucleosome units result in loosely wound chromatin (euchromatin). (**B**) DNA methylation occurs directly on the DNA strand, specifically on the fifth carbon of the cytosine residue, generating 5mC (5-methyl-Cystosine). In mammals, it typically occurs at ‘CpG’ sites, in reference to cytosine-phosphate-guanine dinucleotides. (**C**) Histones form the nucleosome core, which is an octomeric protein complex, formed of four sets of duplicate histones. Approximately 147 bps of DNA wraps around each multi-histone complex to form a nucleosome. The histone tail is the site of histone modifications. Created in BioRender https://BioRender.com/f59j650 (accessed on 24 February 2025) (**A**); https://BioRender.com/b07v215 (accessed on 24 February 2025) (**B**); https://BioRender.com/d17m375 (accessed on 24 February 2025) (**C**).

Epigenetics is defined as mitotically heritable changes to chromatin that alter gene expression without altering DNA sequence. They may occur directly on the DNA strand or on histones and are known as DNA methylation (DNAm) and histone modifications (Figure 1A), respectively.

### 2.1. DNAM

DNAm occurs on the fifth carbon of cytosine residues, creating 5-methylcytosine (5mC; Figure 1B); its addition is mediated by a class of enzymes known as DNA methyltransferases (DNMTs; DNMT1, DNMT2/TRDMT1, DNMT3A, DNMT3B, and DNMT3L) whilst its active removal is mediated by a class of enzymes known as ten-eleven translocation (TETs; TET1, TET2, TET3) proteins. TETs actively demethylate cytosine residues through oxidation, converting 5mC to 5-hydroxymethylcystosine (5hmC) > 5-formylcystosine (5fC) > 5-carboxylcystosine (5caC) [22], after which an enzyme named thymine DNA glycosylate (TDG) restores cytosine [23,24,25]. These different forms have distinct biochemical and biological properties [26,27,28,29], although 5mC and 5hmC cannot be distinguished using common assay methods [30]. Methylated cytosine residues are mitotically heritable, with each daughter cell containing hemi-methylated CpG residues that are recognised by DNMT1 co-factor, Ubiquitin-like, containing PHD and RING finger domains, 1 (UHRF1) to recruit DNMT1 and reinstate CpG methylation [31,32]. The inhibition of DNMT1 has been shown to induce passive demethylation as cells divide without their methylation status being reinstated, and it has been shown that the oxidised forms of 5mC also impede this mechanism [33,34,35]. Therefore, it can be inferred that TET proteins also play a peripheral role in passive demethylation.

### 2.2. Histone Modifications

Histone modifications encompass a broad category of molecular modifications made to the amino acid residues of histone tails. Briefly, a histone is an octomeric protein complex comprising duplicates of four core proteins: histone protein 2A (H2A), histone protein 2B (H2B), histone protein 3 (H3), and histone protein 4 (H4) [36] (Figure 1C). Histones have an overall net positive charge, which facilitates the interaction with DNA that allows for a segment of the DNA strand, approximately 147 base pairs (bps) long [36], to wind around the histone like spun wool wraps around a spool (Figure 1C). DNA wrapped around a histone is referred to as a ‘nucleosome’. Many nucleosomes are referred to as ‘chromatin’, which may be loosely wound (‘euchromatin’) or tightly packed (‘heterochromatin’). The relationship between DNA and histone modifications influencing nucleosome packing to effect chromatin winding, gene accessibility, and subsequent gene expression lies at the crux of the epigenetic modulation of gene expression.

**Figure 2:**
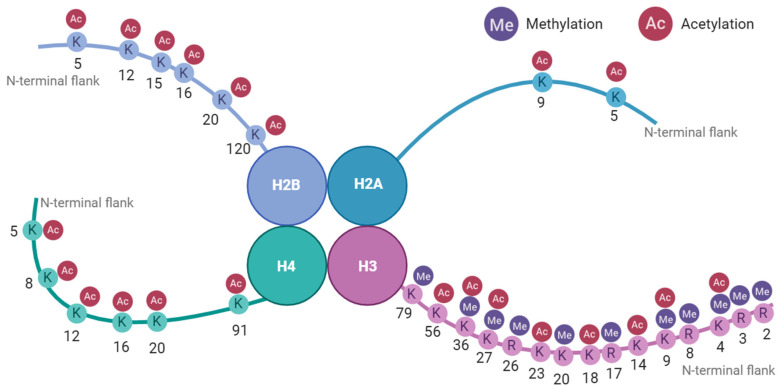
Post-translational histone modifications can occur on each amino acid residue on the respective histone tail. Possible modifications include the addition/removal of an acetyl mark or mono-, di-, or tri-methyl mark. This image schematically represents the location of most well-understood [38,39] acetylation and methylations marks for each histone protein. Created in BioRender https://BioRender.com/t35q375 (accessed on 24 February 2025).

Each histone has an N-terminal region represented by histone ‘tails’. Awareness of the composition of a histone tail is essential to understanding the biochemical interactions underlying epigenetic modulation. A histone tail comprises amino acid residues that are ‘intrinsically disordered’, which means that they have no set 3D structure [37], offering them dynamism and heterogeneity that confers the ability to engage in transient and flexible interactions with multiple binding partners [37]. Histone tails are the sites of post-translational modifications (Figure 2).

Although there are many ways a histone can be post-translationally modified [40] to affect epigenetic modulation, the most well understood is through acetylation or methylation. Histone acetylation is induced by a class of enzymes known as histone acetyltransferases (HATs) [41,42] and removed by histone deacetylases (HDACs) [43]. Histone acetylation is the simpler modification of the two. First, it only occurs on lysine residues and, second, there is only one acetyl moiety per residue. Histone methylation, on the other hand, can occur on either lysine [44,45,46] or arginine [47,48] residues and is induced by lysine methyltransferases (KMTs) [49,50] and protein arginine N-methyltransferases (PRMTs) [51]. In the case of lysine methylation, there may be up to three methyl moieties on one single lysine residue [44,45,46], allowing for mono-, di-, or tri-methylation, whilst an arginine residue can have up to two methyl moieties [47,48], allowing for mono- or di-methylation, with the latter being either symmetrical or asymmetrical [52].

Due to this complexity, a system of nomenclature—known as the ‘Brno nomenclature’ [53]—for histone modifications was established to aid research and communication about the nucleosome. Within the Brno system, each histone modification is referred to by (1) the histone from which the tail protrudes; (2) the amino acid modified, as a single-letter code [54]; (3) the residue number, with numbering starting from the N-terminal flank; and (4) the type of modification, e.g., ‘ac’ = acetylation, ‘me’ = mono-methylation, ‘me3’ = tri-methylation. For example, ‘H3K27ac’ would read as acetylation of the 27th lysine residue on histone 3.

### 2.3. Epigenetic Regulation of Transcription

The known effects of each epigenetic modification on gene transcription are vast and varied. DNAm is the most well-understood mark, particularly when it occurs on CpG islands aligning with gene promoter regions. The most well-understood histone marks include lysine methylation of H3 (K4, K9, K27, K36, K79, and K20) [38]; the arginine methylation of H3 (R2, R8, R17, R26, and R3) [38]; and lysine acetylation of H3 (K4, K9, K14, K18, K23, K27, K36, and K56), H4 (K5, K8, K12, K16, K20, and K91), H2A (K5 and K9), and H2B (K5, K12, K15, K16, K20, and K120) [39] (Figure 2). The preponderance of one histone modification over another is largely attributed to the affinity of enzymes for specific histone residue substrates [55,56,57]. However, research showing that a histone modifying enzyme’s substrate specificity [41,57,58] and activity [59] can be modulated by the protein complex it is associated with points to the intricate complexity involved in these mechanisms.

#### 2.3.1. DNAM Modulation of Gene Expression

It is commonly understood that when DNAm occurs at promoter regions, the methyl moiety interferes with RNA polymerase II’s (RNApol II) recognition of the transcriptional start site, thereby suppressing gene expression [60,61]. Such promoter silencing has been evidenced in germline-specific genes to induce post-implantation somatic differentiation [62,63]. Evidence has shown that other mechanisms through which DNAm suppresses gene expression include interfering with transcription factor binding [61,64] and altering chromatin structure [65]. DNAm does not always suppress gene expression, as it has also been shown to enhance gene expression depending on the genomic context [66,67]. For example, the DNAm of other gene regulatory regions across the gene body, such as enhancers [60] and alternative splice sites [61,65], exerts influence on cis-regulatory “cryptic” and alternative promoter regions. In doing so, DNAm can regulate the nature of the transcript and facilitate transcription across gene bodies [68,69,70]. Determining if the effect of intergenic DNAm on gene upregulation is a consequence of transcriptional elongation or the repression of cryptic promoters remains an active area of research [66], but it remains undoubted that the overall effect of DNAm is to exhibit a lineage specific methylation pattern of each cell’s DNA that helps shape the transcriptional landscape to dictate cell type and behaviour.

#### 2.3.2. Modified Histone Effects on Gene Expression

There is a broad diversity of histone modifications and, as such, histone modifications exert sophisticated effects on transcriptional dynamics. Histone acetylation has been associated with active gene transcription [71] through promoting an open ‘euchromatin’ structure [71]. This is possible because the addition of an acetyl moiety to the lysine residue neutralises its net positive charge [72], thereby reducing the force of electrostatic attraction between the histone and negatively charged DNA strand. Such chromatin remodelling allows the transcriptional machinery access to gene promoter regions, with concordant histone acetylation status shared between promoter and enhancer regions [73]. Other functions of histone acetylation have been shown to include stabilising nucleosome stacking [74] and acting as cellular memory [75] through the recruitment of ‘reader’ [76] proteins such as the bromo- and extra-terminal (BET) domain proteins [77] to direct the post-mitotic transcription of daughter cells. Research has shown that lysine residues most distal from the N-terminal region are tucked into the nucleosome core and play a larger role in nucleosome stability; for example, H3K56ac acts in this way to promote faster DNA unwinding [78]. These examples demonstrate the physicochemical mechanisms through which histone modifications affect transcription.

Histone methylation has been shown to often interact with and affect the prevalence of other epigenetic marks. For example, H3K4me3 recruits the HAT paralog CBP/p300 to facilitate H3K4 acetylation [79], whilst H3K9me3 has been shown to interact with DNAm to silence genes by promoting tightly wound heterochromatin [80]. Research showing UHRF1 docking modulated by histone modifications demonstrates that post-translational modifications of histones play a role in DNAm modulation through development [81,82].

Considering the varying effects of a single epigenetic mark, it is evident that the biochemical nature of the mark is only one piece of the puzzle. Epigenetic marks have been shown to interact with one another to affect transcriptional dynamics, chromatin winding, and gene expression [83,84,85]. Other factors that have been shown to play a role heavily depend on the genomic location of the epigenetic mark, thereby modulating its cis-regulatory effect and which regions are affected. It must be noted that the information provided above gives a broad understanding of histone modifications but the specific positioning of each residue and how they interact with one another—also known as the ‘histone code’ [86]—is an intricate, important, and rapidly growing area of research that can be conceptualised in terms of how the modified residues interact to dictate a chromatin state [87].

## 3. DNAM During Mammalian Development

**Figure 3:**
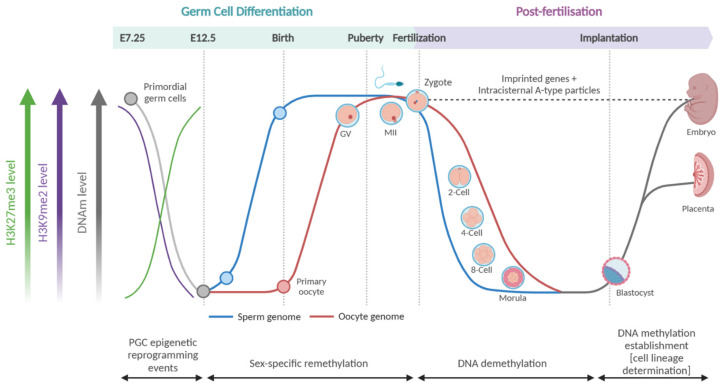
Epigenetic reprogramming events during germ cell differentiation and post-fertilisation: these represent the two largest epigenetic reprogramming events in mammalian development. Mammals undergo global DNA demethylation during these stages, punctuated by sex- and lineage-specific re-methylation events. More recent evidence shows that the prevalence of histone modifications, such as H3K27me3 and H3K9me2, also fluctuates during PGC differentiation [92,143,144]. Created in BioRender https://BioRender.com/f90v429 (accessed on 24 February 2025).

In mammals, it has long been accepted that there are two major waves of epigenetic reprogramming during development, namely, (1) post-fertilisation and (2) during gametogenesis [88] (Figure 3), and technological innovation has allowed for this to be temporally visualised [89,90,91]. During embryogenesis, it has been shown that sites of DNAm from both the maternal and paternal lines are actively demethylated post-fertilisation [89] to ensure a balanced DNAm dosage across maternal and paternal alleles, with the blastocyst expressing DNAm levels as low as 20% (Figure 3). Interestingly, residual DNAm predominantly resides on genomic imprints and some transposable element (TE) orders [89,90]. Following implantation, DNMTs work to restore DNAm levels to 70–80% in a pattern-specific manner maintained in somatic cells [63,92,93]. This pattern of demethylation also occurs in primordial germ cells (PGCs) as they commence expansion and migration within the developing organism, with the only difference being that once the PGCs migrate across the posterior embryonic ridge to enter the gonads, they also undergo demethylation of imprinted alleles, CpG islands on the X-chromosome, and germline-specific genes, followed by the reestablishment of DNAm in a sex-specific manner [94,95,96].

### 3.1. Germ Cell Differentiation

In the prenatal organism, the male and female PGCs have similar CpG methylation levels (approximately 20–30%) [97]. This hypomethylated status is induced by an initial global passive demethylation event, followed by the TET-dependant demethylation of imprinting control regions and meiotic genes [98]. In the post-natal organism, the resultant female gonadal cell has been shown to be hypomethylated relative to somatic cells; for example, murine and human oocytes express approximately 20–50% DNAm, whilst somatic cells express 70–80% [99,100,101]. Sperm cells, however, exhibit global CpG methylation levels from 50 to 90% [102,103,104]. The establishment of gametic methylation is mediated by DNMT3L and DNMT3A [105,106] and works to maintain cell lineage, the integrity of genomic imprints, successful fertilisation, and embryonic development. Therefore, any induced aberrations in gamete DNAm may serve as a source of error transmitted to the zygote.

Male PGCs and sperm undergo several windows of susceptibility to the induction of DNAm aberrations: first, prenatally around gestational week 8, then postnatally during the first 4 months after birth, during peri-puberty (~9 years old), and puberty (~11 years old) [107]. On the other hand, foetal oogonia remain arrested at the first meiotic division until puberty wherein meiosis II occurs to develop fully grown oocytes during ovulation [108], with DNAm being predominantly reestablished at later stages of follicular development [100]. This sex-specific divergence of gametes leads to a pronounced difference in DNAm levels for spermatogonia and oogonia at birth, being hyper- and hypomethylated, respectively.

Studies have shown variation in gametic CpG methylation caused by exposure to environmental pollutants, and comprehensive reviews for sperm [107] and oocytes [109] have been published in this area. Some highlighted examples include research showing that nicotine and cannabis exposure (both alone and combined) alters sperm DNAm [102,103,104,110,111,112], with gene set enrichment analysis showing that these changes occur along important neurodevelopmental genes [113]. Similarly, air pollution has also been shown to affect sperm DNAm globally [114,115] and in genes enriched for neurodevelopmental pathways [116], with associations to lower neonatal birth weight [117]. Exposure to other environmental pollutants, such as phthalate, has been shown to alter sperm DNAm in a manner associated with lower sperm motility [118] and blastocyst quality [119,120].

### 3.2. Post-Fertilisation

Upon fertilisation of the fully differentiated oocyte, the gametic nuclei fuse to form the zygote. It is now that the differentiated gametes are reprogrammed to undifferentiated embryonic cells in the totipotent state. Initially, the zygotic genome is not expressed, and reprogramming is dependent on maternal RNAs and proteins supplied by the oocyte [121]. After this, maternal-to-zygotic (MZT) genome expression occurs and gives way to a process known as zygotic genome activation (ZGA) [121]. Upon successful formation of polar bodies, the first cell division occurs to generate a two-cell embryo. Successive embryonic cell divisions occur until we have a 32-cell morula, followed by the blastocyst stage of embryonic development. A blastocyst comprises embryonic cells that have differentiated to form the inner cell mass (ICM) and trophoectoderm, which go on to form the foetus and the placenta, respectively [122,123].

Early embryonic cells (two-cell to morula stage) have an unspecified lineage and are therefore capable of differentiating into any cell necessary to grow a new organism. The term pointing to this broad potential is ‘totipotency’ [124,125]. It is worth noting that as technology allows us to delineate further mechanisms, a stricter usage of the term ‘totipotency’ limited to the one-to-two-cell stages [126] has been posited but, for the purposes of this review, we will apply the term to the two-cell to morula stage. Embryonic cells at the blastocyst stage have diverged to lose totipotency. The cells in the ICM are now referred to as pluripotent, with potential to differentiate into any cell type within the body, but they cannot independently form extraembryonic tissue. Interestingly, the levels of DNAm within human embryos sharply decrease during ZGA and steadily drop until embryonic implantation [90,127]. Looking at the contribution of the male and female germ lines to the preimplantation embryonic DNAm landscape shows that measured embryonic demethylation is largely attributed to the paternal genome [128,129], whilst germ-line factor DPPA3 works to abrogate DNMT1-mediated oocyte demethylation [130]. Single-cell resolution DNAm sequencing of the human embryo confirms the demethylation of the preimplantation embryo and validates the idea that the DNAm decrease is owed to the paternal germline [90].

Upon embryonic implantation, DNMT3b is predominantly initiated [131] to cause a sharp increase in DNAm [127]. Re-methylation works to establish the somatic DNAm pattern necessary to facilitate cell differentiation into lineages that will form the tissue types central to foetal growth and development [132]. Gastrulation marks the commencement of development towards a multilayered and dimensional organism comprising cells at different stages on their hierarchical journey towards differentiation. It has been shown that the reestablishment of DNAm during this time is heavily underpinned by the activity of DNMT3A and DNMT3B [67,133].

## 4. Histone Modifications During Mammalian Development

### 4.1. Post-Fertilisation

When considering the parental contributions to the epigenetic state of the embryo, it is important to note that paternal chromatin does not make a large contribution toward the embryonic histones, due to the way in which chromatin is packed within sperm. As spermatids undergo spermiogenesis, the histone proteins are ejected in favour of protamines, which allows for the denser compaction of the paternal genome into the spermatozoon head [134], with only a small fraction of histones remaining [135]. As such, histones in the developing embryo are predominantly obtained from the maternal chromatin [136] or are newly synthesised. Although paternal histone contribution cannot be overlooked, the exact extent of contribution remains to be elucidated. Parental histones become reinstated with high spatial fidelity during replication [137] with the assistance of histone chaperone proteins, DNA polymerase, Mcm2, Ctf4, and Polα to ensure symmetry between leading and lagging nascent strands [138,139,140]. This important mechanism ensures faithful transcription in the daughter cell, with research showing that asymmetry causes the loss of silencing in a TE family known as endogenous retroviruses (ERVs) [140].

### 4.2. Primordial Germ Cell Differentiation

As murine PGCs experience global demethylation, a concomitant depletion and enrichment of H3K9me2 and H3K27me3, respectively, has been shown [94,141,142] (Figure 3), with a proportion of the marks occurring in replacement of DNAm and the remaining occurring de novo, coupled with other repressive histone marks such as H3K9me3. Histone modifications play an important role in developmental gene regulation in a manner separate to DNAm, with evidence [143] using murine embryonic stem cells showing that the deletion of H3K9me3 leads to the initiation of genes different to those caused by abrogating DNMT function, with most notable effect at TEs. The in vitro modelling of human PGCs [144] showed that H3K9me2 decreased post-PGC migration compared to somatic cells, but did not deplete to the same extent as murine PGCs, and H3K27me3 enrichment was to a lesser magnitude and shorter duration in human PGCs compared to mouse. This demonstrates that although both mammalian cell types undergo changes in histone modifications during development, H3K9me3 and H3K27me3 express unique temporal dynamics between species. This pattern was also detected using Western blotting and immunofluorescence [142]. Interestingly, it was also shown [144] that areas resistant to demethylation, such as TEs, were enriched for H3K9me3, KAP1, and ZFP57, pointing to a role for a KAP1/ZFP57 complex interaction with H3K9me3 to maintain or reinstate methylation, although the exact mechanistic role remains to be elucidated. Human PGCs have showed an increase in active histone modifications H3K27ac and H3K4me3 and a decrease in repressive mark H3K27me3 along TEs compared to somatic germ cells [142], with H3K9me3 more enriched along evolutionary younger elements.

The above-mentioned findings point to an important role of histone modifications to modulate transcription during broad DNA demethylation events that occur during embryonic development. What is further highlighted is that aberrations in the newly developed organism’s histone code effect TEs, of which some families are largely reported to escape DNA demethylation events, thereby suggesting that the role of histone modifications in development are not simply to compensate for global DNA demethylation events. These findings warrant further investigation into the role of histone modifications during development.

## 5. Mammalian Epigenetic Transmission

Epigenetic marks are heritable by definition but this cellular heritability can occur in two ways: (1) ‘replicative’ where the transmitted epigenetic marks are maintained through meiosis as they are through mitosis; or (2) ‘restorative’ where the transmitted primary epigenetic marks have been erased but then reestablished with high fidelity to the template. In the context of an intergenerational epigenetic mark, restorative transmission would occur during gametogenesis and embryonic development, whilst replicative transmission would contribute to cell lineage determination and differentiation, but this remains to be inextricably defined to date. This section outlines the latest research on what we know about cellular epigenetic heritability.

### 5.1. Replicative Maintenance

In the context of DNAm, replicative maintenance is mediated by DNMT1 [145]—the ‘maintenance DNA methyltransferase’—as per the mechanism described in Section 2.1 above. Histone modifications are also maintained during DNA replication through the redistribution of modified histones between template and nascent strands, as has been shown in human [146] and mouse [147] cells, resulting in the high-fidelity of chromatin domains between parent and daughter cells [148,149]. It has been shown that specific histone modifications, such as H3K27ac [150] and H4K5ac [145], are not erased during mitosis and therefore act as ‘bookmarks’ to assist in post-replicative transcriptional fidelity in the daughter cell with the assistance of a transcription factor, such as BRD4 [145].

### 5.2. Restorative Maintenance

There is strong evidence for the restorative maintenance of CpG methylation in the germline and embryo [89,94], yet the mechanism driving site-specific DNAm reestablishment remains to be elucidated. The role of a ‘bookmark’ signal has been posited, and studies showing the modulatory effect of piwi RNAs [151,152] and transcription factors [153] on DNAm levels in mammals have been published. Interestingly, Festuccia et al. [154] point to evidence [155] showing cellular differentiation taking place after mitosis with an extended G1 phase, and posit that bookmarking mechanisms similar to those proven in mitosis might also play a role in the epigenetic determination of cell lineage. To date, no single mechanism has been shown to act as the primary driver of DNAm memory in mammals.

However, this does not mean that epigenetic inheritance in mammals is not shown. The best example of this is the phenomenon of ‘metastable epialleles’ as demonstrated by the *Agouti viable yellow (A^vy^)* and *Axin fused (Axin^Fu^)* alleles in mice [156,157,158,159], wherein a spontaneous insertion of a class II TE, intracisternal A-particle (IAP), in the *Agouti* coat colour gene locus resulted in an observable phenotypic difference (in coat colour) modulated by DNAm [158,160,161]. This is made feasible through IAPs being shown to evade the widespread demethylation events occurring during development [89], thereby allowing for a mode of intergenerational epigenetic inheritance in mammals until the F2 generation.

Studies have demonstrated intergenerational phenotypic inheritance in mammals up until the F2 generation, such as the effect of paternal obesity on offspring adiposity [162] and gestational diabetes mellitus on increased chronic disease prevalence in offspring [163,164]. The link between the propagation of a biological phenotype induced by environmental exposure is supported by evidence of altered DNAm in the F1 generation associated with gestational diabetes mellitus [165,166], prenatal nutrition deficit [167], maternal age [168], and mental health [169] in humans.

Transgenerational epigenetic inheritance (beyond the F2 generation) has been shown extensively in animals such as zebrafish [170] and *Caenorhabditis elegans* [171], but the mechanisms are less well understood in mammals. However, prenatal exposure to harsh chemicals, such as the agricultural fungicide vinclozolin, has been shown to induce DNAm changes at the F3 generation [172].

## 6. DNAM of TEs During Development

The measured global DNA demethylation waves reported above do not affect all genomic regions equally and some regions, such as active repeat elements and IAPs, escape these demethylation events [90]. However, it should be noted that the younger retroelements, such as Long Interspersed Nuclear Elements (LINEs), Short Interspersed Nuclear Elements (SINEs), and ERVs, are demethylated in the developmental demethylation waves described above [89,90]. Delineating the contribution of these TEs to the development and long-term health span of the offspring is of great interest. For example, TEs have been shown to contribute to transcriptome activation from as early as the two-cell stage [173]. Interestingly it is a demethylated TE order, murine ERVs, that contributes to this effect and not the continuously methylated IAPs [173], thereby demonstrating the transcriptional control exerted by DNAm on TE expression throughout development.

**Figure 4:**
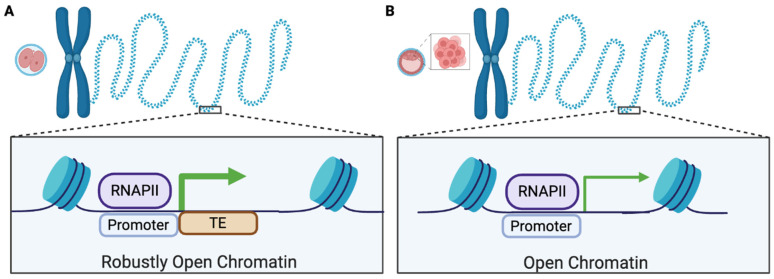
TEs enhance chromatin unwinding and ZGA gene transcription. (**A**) Early-stage embryos have been shown to express enhanced chromatin unwinding [165] and gene expression [172] (denoted by green arrow) at locations corresponding with demethylated TEs [167], compared to (**B**) ICM cells. Created in BioRender. Zakarya, R. (2025) https://BioRender.com/x86b757 (accessed on 24 February 2025).

Another mechanism through which TEs exert their effect during development is by acting as “cryptic promoters”, wherein those TE classes that have been demethylated in the preimplantation embryo will be co-opted by ZGA genes to act as gene enhancer regions in the early developing embryo [174,175,176] (Figure 4), thereby affecting chromatin unwinding and gene expression at the early stages of development. This demonstrates a stunning capacity of TEs to affect gene expression when demethylated and places great import on the fidelity of restorative DNAm of the blastocyst and post-implantation.

## 7. Conclusions

Epigenetic changes have been shown in patients with many chronic health conditions, such as asthma [177], metabolic disease [178,179,180], and heart disease [180]. Although associations with environmental exposures have been made, the etiological molecular mark connecting the deleterious environment with disease onset and progression has yet to be elucidated. The DOHaD hypothesis posits that epigenetic marks upon lineage specification confers the mechanistic basis of these chronic diseases. In this review, we have summarised the broad field of epigenetics to highlight potential mechanisms through which developmental epigenetic insult may be conferred to the offspring. Future studies of parental contribution and the developmental environment on the health of the offspring should consider the intersection between these mechanisms to best address the growing health challenge posed by chronic disease.

## Data Availability

Not applicable.

## Abbreviations

The following abbreviations are used in this manuscript:

5caC	5-carboxymethylcytosine
5fc	5-formylmethylcystosine
5hmC	5-hydroxymethylcytosine
5mC	5-methylcytosine
BET	bromo- and extra-terminal
bps	base pairs
DNAm	DNA methylation
DNMT	DNA methyltransferase
DOHaD	Developmental Origins of Health and Disease
ERV	endogenous retrovirus
H2A	histone 2A
H2B	histone 2B
H3	histone 3
H4	histone 4
HAT	histone acetyltransferase
HDAC	histone deacetylase
IAP	intracisternal-A particle
ICM	inner cell mass
KMT	lysine methyltransferase
LINE	long interspersed nuclear element
MZT	maternal to zygotic transcription
PGCs	primordial germ cells
PRMT	protein arginine N-methyltransferase
RNA Pol II	RNA polymerase II
SINE	short interspersed nuclear element
TDG	thymine DNA glycosylate
TE	transposable element
TET	ten-eleven translocation
UHRF1	ubiquitin-like, containing PHD and RING finger domains, 1
ZGA	zygotic genome activation

## References

[1] Vos T., Lim S.S., Abbafati C., Abbas K.M., Abbasi M., Abbasifard M., Abbasi-Kangevari M., Abbastabar H., Abd-Allah F., Abdelalim A. (2020). Global burden of 369 diseases and injuries in 204 countries and territories, 1990–2019: A systematic analysis for the Global Burden of Disease Study 2019. Lancet.

[2] Hacker K. (2024). The burden of chronic disease. Mayo Clin. Proc. Innov. Qual. Outcomes.

[3] Countdown N. (2018). NCD Countdown 2030: Worldwide trends in non-communicable disease mortality and progress towards Sustainable Development Goal target 3.4. Lancet.

[4] Murray C.J.L. (2022). The Global Burden of Disease Study at 30 years. Nat. Med..

[5] Han Y., Jia Q., Jahani P.S., Hurrell B.P., Pan C., Huang P., Gukasyan J., Woodward N.C., Eskin E., Gilliland F.D. (2020). Genome-wide analysis highlights contribution of immune system pathways to the genetic architecture of asthma. Nat. Commun..

[6] Eichler E.E., Flint J., Gibson G., Kong A., Leal S.M., Moore J.H., Nadeau J.H. (2010). Missing heritability and strategies for finding the underlying causes of complex disease. Nat. Rev. Genet..

[7] Kim K.W., Ober C. (2019). Lessons Learned From GWAS of Asthma. Allergy, Asthma Immunol. Res..

[8] Weiss S.T., Silverman E.K. (2011). Pro: Genome-Wide Association Studies (GWAS) in Asthma. Am. J. Respir. Crit. Care Med..

[9] Pasipoularides A. (2015). Linking Genes to Cardiovascular Diseases: Gene Action and Gene–Environment Interactions. J. Cardiovasc. Transl. Res..

[10] Von Mutius E. (2009). Gene-environment interactions in asthma. J. Allergy Clin. Immunol..

[11] Goyal D., Limesand S.W., Goyal R. (2019). Epigenetic responses and the developmental origins of health and disease. J. Endocrinol..

[12] Haugen A.C., Schug T.T., Collman G., Heindel J.J. (2014). Evolution of DOHaD: The impact of environmental health sciences. J. Dev. Orig. Health Dis..

[13] Lacagnina S. (2019). The Developmental Origins of Health and Disease (DOHaD). Am. J. Lifestyle Med..

[14] Waterland R.A., Michels K.B. (2007). Epigenetic Epidemiology of the Developmental Origins Hypothesis. Annu. Rev. Nutr..

[15] Lite C., Raja G.L., Juliet M., Sridhar V.V., Subhashree K.D., Kumar P., Chakraborty P., Arockiaraj J. (2022). In utero exposure to endocrine-disrupting chemicals, maternal factors and alterations in the epigenetic landscape underlying later-life health effects. Environ. Toxicol. Pharmacol..

[16] Deodati A., Inzaghi E., Cianfarani S. (2020). Epigenetics and In Utero Acquired Predisposition to Metabolic Disease. Front. Genet..

[17] Oken E., Huh S.Y., Taveras E.M., Rich-Edwards J.W., Gillman M.W. (2005). Associations of Maternal Prenatal Smoking with Child Adiposity and Blood Pressure. Obes. Res..

[18] Zhang B., Liang S., Zhao J., Qian Z., A Bassig B., Yang R., Zhang Y., Hu K., Xu S., Zheng T. (2016). Maternal exposure to air pollutant PM2.5 and PM10 during pregnancy and risk of congenital heart defects. J. Expo. Sci. Environ. Epidemiol..

[19] Ong T.P., Guest P.C. (2018). Nutritional programming effects on development of metabolic disorders in later life. Investigations of Early Nutrition Effects on Long-Term Health: Methods and Applications.

[20] Lu C., Wang L., Liao H., Li B., Liu Q., Wang F. (2023). Impacts of intrauterine and postnatal exposure to air pollution on preschool children’s asthma: A key role in cumulative exposure. Build. Environ..

[21] Sunde R.B., Thorsen J., Pedersen C.-E.T., Stokholm J., Bønnelykke K., Chawes B., Bisgaard H. (2021). Prenatal tobacco exposure and risk of asthma and allergy outcomes in childhood. Eur. Respir. J..

[22] Kohli R.M., Zhang Y. (2013). TET enzymes, TDG and the dynamics of DNA demethylation. Nature.

[23] Kriaucionis S., Heintz N. (2009). The Nuclear DNA Base 5-Hydroxymethylcytosine Is Present in Purkinje Neurons and the Brain. Science.

[24] Weber A.R., Krawczyk C., Robertson A.B., Kuśnierczyk A., Vågbø C.B., Schuermann D., Klungland A., Schär P. (2016). Biochemical reconstitution of TET1–TDG–BER-dependent active DNA demethylation reveals a highly coordinated mechanism. Nat. Commun..

[25] Maiti A., Drohat A.C. (2011). Thymine DNA glycosylase can rapidly excise 5-formylcytosine and 5-carboxylcytosine: Potential implications for active demethylation of CpG sites. J. Biol. Chem..

[26] Ehrlich M., Ehrlich K.C. (2014). DNA Cytosine Methylation and Hydroxymethylation at the Borders. Epigenomics.

[27] Lister R., Mukamel E.A., Nery J.R., Urich M., Puddifoot C.A., Johnson N.D., Lucero J., Huang Y., Dwork A.J., Schultz M.D. (2013). Global Epigenomic Reconfiguration During Mammalian Brain Development. Science.

[28] Li L., Gao Y., Wu Q., Cheng A.S., Yip K.Y. (2019). New guidelines for DNA methylome studies regarding 5-hydroxymethylcytosine for understanding transcriptional regulation. Genome Res..

[29] Spruijt C.G., Gnerlich F., Smits A.H., Pfaffeneder T., Jansen P.W.T.C., Bauer C., Munzel M., Wagner M., Muller M., Khan F. (2013). Dynamic readers for 5-(hydroxy) methylcytosine and its oxidized derivatives. Cell.

[30] Nestor C., Ruzov A., Meehan R.R., Dunican D.S. (2010). Enzymatic Approaches and Bisulfite Sequencing Cannot Distinguish Between 5-Methylcytosine and 5-Hydroxymethylcytosine in DNA. BioTechniques.

[31] Bostick M., Kim J.K., Estève P.-O., Clark A., Pradhan S., Jacobsen S.E. (2007). UHRF1 Plays a Role in Maintaining DNA Methylation in Mammalian Cells. Science.

[32] Sharif J., Muto M., Takebayashi S.-I., Suetake I., Iwamatsu A., Endo T.A., Shinga J., Mizutani-Koseki Y., Toyoda T., Okamura K. (2007). The SRA protein Np95 mediates epigenetic inheritance by recruiting Dnmt1 to methylated DNA. Nature.

[33] Hashimoto H., Liu Y., Upadhyay A.K., Chang Y., Howerton S.B., Vertino P.M., Zhang X., Cheng X. (2012). Recognition and potential mechanisms for replication and erasure of cytosine hydroxymethylation. Nucleic Acids Res..

[34] Ji D., Lin K., Song J., Wang Y. (2014). Effects of Tet-induced oxidation products of 5-methylcytosine on Dnmt1- and DNMT3a-mediated cytosine methylation. Mol. Biosyst..

[35] Otani J., Kimura H., Sharif J., Endo T.A., Mishima Y., Kawakami T., Koseki H., Shirakawa M., Suetake I., Tajima S. (2013). Cell cycle-dependent turnover of 5-hydroxymethyl cytosine in mouse embryonic stem cells. PLoS ONE.

[36] Luger K., Mäder A.W., Richmond R.K., Sargent D.F., Richmond T.J. (1997). Crystal structure of the nucleosome core particle at 2.8 Å resolution. Nature.

[37] Uversky V.N. (2014). Introduction to Intrinsically Disordered Proteins (IDPs). Chem. Rev..

[38] Greer E.L., Shi Y. (2012). Histone methylation: A dynamic mark in health, disease and inheritance. Nat. Rev. Genet..

[39] A Musselman C., Lalonde M.-E., Côté J., Kutateladze T.G. (2012). Perceiving the epigenetic landscape through histone readers. Nat. Struct. Mol. Biol..

[40] Tan M., Luo H., Lee S., Jin F., Yang J.S., Montellier E., Buchou T., Cheng Z., Rousseaux S., Rajagopal N. (2011). Identification of 67 Histone Marks and Histone Lysine Crotonylation as a New Type of Histone Modification. Cell.

[41] Radzisheuskaya A., Shliaha P.V., Grinev V.V., Shlyueva D., Damhofer H., Koche R., Gorshkov V., Kovalchuk S., Zhan Y., Rodriguez K.L. (2021). Complex-dependent histone acetyltransferase activity of KAT8 determines its role in transcription and cellular homeostasis. Mol. Cell.

[42] Poziello A., Nebbioso A., Stunnenberg H.G., Martens J.H., Carafa V., Altucci L. (2020). Recent insights into *Histone Acetyltransferase-1*: Biological function and involvement in pathogenesis. Epigenetics.

[43] Park S.-Y., Kim J.-S. (2020). A short guide to histone deacetylases including recent progress on class II enzymes. Exp. Mol. Med..

[44] Murray K. (1964). The Occurrence of iε-N-Methyl Lysine in Histones. Biochemistry.

[45] Paik W.K., Kim S. (1967). E-N-dimethyllysine in histones. Biochem. Biophys. Res. Commun..

[46] Hempel K., Lange H.W., Birkofer L. (1968). Epsilon-N-trimethyllysine, a new amino acid in histones. Naturwissenschaften.

[47] Byvoet P., Shepherd G., Hardin J., Noland B. (1972). The distribution and turnover of labeled methyl groups in histone fractions of cultured mammalian cells. Arch. Biochem. Biophys..

[48] Borun T.W., Pearson D., Paik W.K. (1972). Studies of Histone Methylation during the HeLa S-3 Cell Cycle. J. Biol. Chem..

[49] Feng Q., Wang H., Ng H.H., Erdjument-Bromage H., Tempst P., Struhl K., Zhang Y. (2002). Methylation of H3-Lysine 79 Is Mediated by a New Family of HMTases without a SET Domain. Curr. Biol..

[50] Rea S., Eisenhaber F., O’Carroll D., Strahl B.D., Sun Z.-W., Schmid M., Opravil S., Mechtler K., Ponting C.P., Allis C.D. (2000). Regulation of chromatin structure by site-specific histone H3 methyltransferases. Nature.

[51] Bannister A.J., Kouzarides T. (2011). Regulation of chromatin by histone modifications. Cell Res..

[52] Litt M., Qiu Y., Huang S. (2009). Histone arginine methylations: Their roles in chromatin dynamics and transcriptional regulation. Biosci. Rep..

[53] Turner B.M. (2005). Reading signals on the nucleosome with a new nomenclature for modified histones. Nat. Struct. Mol. Biol..

[54] Swanson R. (1984). A unifying concept for the amino acid code. Bull. Math. Biol..

[55] Hansen B.K., Gupta R., Baldus L., Lyon D., Narita T., Lammers M., Choudhary C., Weinert B.T. (2019). Analysis of human acetylation stoichiometry defines mechanistic constraints on protein regulation. Nat. Commun..

[56] Weinert B.T., Narita T., Satpathy S., Srinivasan B., Hansen B.K., Schölz C., Hamilton W.B., Zucconi B.E., Wang W.W., Liu W.R. (2018). Time-Resolved Analysis Reveals Rapid Dynamics and Broad Scope of the CBP/p300 Acetylome. Cell.

[57] Feller C., Forné I., Imhof A., Becker P.B. (2015). Global and Specific Responses of the Histone Acetylome to Systematic Perturbation. Mol. Cell.

[58] Cai Y., Jin J., Swanson S.K., Cole M.D., Choi S.H., Florens L., Washburn M.P., Conaway J.W., Conaway R.C. (2010). Subunit Composition and Substrate Specificity of a MOF-containing Histone Acetyltransferase Distinct from the Male-specific Lethal (MSL) Complex. J. Biol. Chem..

[59] Wang Z., Millard C.J., Lin C.-L., E Gurnett J., Wu M., Lee K., Fairall L., Schwabe J.W., A Cole P., Brigham (2020). Diverse nucleosome Site-Selectivity among histone deacetylase complexes. eLife.

[60] Baribault C., Ehrlich K.C., Ponnaluri V.K.C., Pradhan S., Lacey M., Ehrlich M. (2018). Developmentally linked human DNA hypermethylation is associated with down-modulation, repression, and upregulation of transcription. Epigenetics.

[61] Shukla S., Kavak E., Gregory M., Imashimizu M., Shutinoski B., Kashlev M., Oberdoerffer P., Sandberg R., Oberdoerffer S. (2011). CTCF-promoted RNA polymerase II pausing links DNA methylation to splicing. Nature.

[62] Auclair G., Guibert S., Bender A., Weber M. (2014). Ontogeny of CpG island methylation and specificity of DNMT3 methyltransferases during embryonic development in the mouse. Genome Biol..

[63] Borgel J., Guibert S., Li Y., Chiba H., Schübeler D., Sasaki H., Forné T., Weber M. (2010). Targets and dynamics of promoter DNA methylation during early mouse development. Nat. Genet..

[64] Yin Y., Morgunova E., Jolma A., Kaasinen E., Sahu B., Khund-Sayeed S., Das P.K., Kivioja T., Dave K., Zhong F. (2017). Impact of cytosine methylation on DNA binding specificities of human transcription factors. Science.

[65] Chandra S., Baribault C., Lacey M., Ehrlich M. (2014). Myogenic Differential Methylation: Diverse Associations with Chromatin Structure. Biology.

[66] Greenberg M.V.C., Bourc’His D. (2019). The diverse roles of DNA methylation in mammalian development and disease. Nat. Rev. Mol. Cell Biol..

[67] Jones P.A. (2012). Functions of DNA methylation: Islands, start sites, gene bodies and beyond. Nat. Rev. Genet..

[68] Neri F., Rapelli S., Krepelova A., Incarnato D., Parlato C., Basile G., Maldotti M., Anselmi F., Oliviero S. (2017). Intragenic DNA methylation prevents spurious transcription initiation. Nature.

[69] Lister R., Pelizzola M., Dowen R.H., Hawkins R.D., Hon G., Tonti-Filippini J., Nery J.R., Lee L., Ye Z., Ngo Q.-M. (2009). Human DNA methylomes at base resolution show widespread epigenomic differences. Nature.

[70] Varley K.E., Gertz J., Bowling K.M., Parker S.L., Reddy T.E., Pauli-Behn F., Cross M.K., Williams B.A., Stamatoyannopoulos J.A., Crawford G.E. (2013). Dynamic DNA methylation across diverse human cell lines and tissues. Genome Res..

[71] Stasevich T.J., Hayashi-Takanaka Y., Sato Y., Maehara K., Ohkawa Y., Sakata-Sogawa K., Tokunaga M., Nagase T., Nozaki N., McNally J.G. (2014). Regulation of RNA polymerase II activation by histone acetylation in single living cells. Nature.

[72] Dancy B.M., Cole P.A. (2015). Protein Lysine Acetylation by p300/CBP. Chem. Rev..

[73] Andersson R., Sandelin A. (2019). Determinants of enhancer and promoter activities of regulatory elements. Nat. Rev. Genet..

[74] Saurabh S., Glaser M.A., Lansac Y., Maiti P.K. (2016). Atomistic simulation of stacked nucleosome core particles: Tail bridging, the H4 tail, and effect of hydrophobic forces. J. Phys. Chem. B.

[75] Strahl B.D., Allis C.D. (2000). The language of covalent histone modifications. Nature.

[76] Lee K.K., Workman J.L. (2007). Histone acetyltransferase complexes: One size doesn’t fit all. Nat. Rev. Mol. Cell Biol..

[77] Jain A.K., Barton M.C. (2017). Bromodomain Histone Readers and Cancer. J. Mol. Biol..

[78] North J.A., Shimko J.C., Javaid S., Mooney A.M., Shoffner M.A., Rose S.D., Bundschuh R., Fishel R., Ottesen J.J., Poirier M.G. (2012). Regulation of the nucleosome unwrapping rate controls DNA accessibility. Nucleic Acids Res..

[79] Crump N.T., Hazzalin C.A., Bowers E.M., Alani R.M., Cole P.A., Mahadevan L.C. (2011). Dynamic acetylation of all lysine-4 trimethylated histone H3 is evolutionarily conserved and mediated by p300/CBP. Proc. Natl. Acad. Sci. USA.

[80] Rottach A., Frauer C., Pichler G., Bonapace I.M., Spada F., Leonhardt H. (2009). The multi-domain protein Np95 connects DNA methylation and histone modification. Nucleic Acids Res..

[81] Rothbart S.B., Dickson B.M., Ong M.S., Krajewski K., Houliston S., Kireev D.B., Arrowsmith C.H., Strahl B.D. (2013). Multivalent histone engagement by the linked tandem Tudor and PHD domains of UHRF1 is required for the epigenetic inheritance of DNA methylation. Genes Dev..

[82] Liu X., Gao Q., Li P., Zhao Q., Zhang J., Li J., Koseki H., Wong J. (2013). UHRF1 targets DNMT1 for DNA methylation through cooperative binding of hemi-methylated DNA and methylated H3K9. Nat. Commun..

[83] Atlasi Y., Stunnenberg H.G. (2017). The interplay of epigenetic marks during stem cell differentiation and development. Nat. Rev. Genet..

[84] Shlyueva D., Stampfel G., Stark A. (2014). Transcriptional enhancers: From properties to genome-wide predictions. Nat. Rev. Genet..

[85] Harmston N., Lenhard B. (2013). Chromatin and epigenetic features of long-range gene regulation. Nucleic Acids Res..

[86] Jenuwein T., Allis C.D. (2001). Translating the Histone Code. Science.

[87] Ernst J., Kellis M. (2010). Discovery and characterization of chromatin states for systematic annotation of the human genome. Nat. Biotechnol..

[88] Monk M., Boubelik M., Lehnert S. (1987). Temporal and regional changes in DNA methylation in the embryonic, extraembryonic and germ cell lineages during mouse embryo development. Development.

[89] Wang L., Zhang J., Duan J., Gao X., Zhu W., Lu X., Yang L., Zhang J., Li G., Ci W. (2014). Programming and inheritance of parental DNA methylomes in mammals. Cell.

[90] Zhu P., Guo H., Ren Y., Hou Y., Dong J., Li R., Lian Y., Fan X., Hu B., Gao Y. (2017). Single-cell DNA methylome sequencing of human preimplantation embryos. Nat. Genet..

[91] Grosswendt S., Kretzmer H., Smith Z.D., Kumar A.S., Hetzel S., Wittler L., Klages S., Timmermann B., Mukherji S., Meissner A. (2020). Epigenetic regulator function through mouse gastrulation. Nature.

[92] Zhang Y., Xiang Y., Yin Q., Du Z., Peng X., Wang Q., Fidalgo M., Xia W., Li Y., Zhao Z.-A. (2017). Dynamic epigenomic landscapes during early lineage specification in mouse embryos. Nat. Genet..

[93] Smith Z.D., Shi J., Gu H., Donaghey J., Clement K., Cacchiarelli D., Gnirke A., Michor F., Meissner A. (2017). Epigenetic restriction of extraembryonic lineages mirrors the somatic transition to cancer. Nature.

[94] Seisenberger S., Andrews S., Krueger F., Arand J., Walter J., Santos F., Popp C., Thienpont B., Dean W., Reik W. (2012). The Dynamics of Genome-wide DNA Methylation Reprogramming in Mouse Primordial Germ Cells. Mol. Cell.

[95] Hajkova P., Erhardt S., Lane N., Haaf T., El-Maarri O., Reik W., Walter J., Surani M. (2002). Epigenetic reprogramming in mouse primordial germ cells. Mech. Dev..

[96] Lee J., Inoue K., Ono R., Ogonuki N., Kohda T., Kaneko-Ishino T., Ogura A., Ishino F. (2002). Erasing genomic imprinting memory in mouse clone embryos produced from day 11.5 primordial germ cells. Development.

[97] Gkountela S., Zhang K.X., Shafiq T.A., Liao W.-W., Hargan-Calvopiña J., Chen P.-Y., Clark A.T. (2015). DNA Demethylation Dynamics in the Human Prenatal Germline. Cell.

[98] Hargan-Calvopina J., Taylor S., Cook H., Hu Z., Lee S.A., Yen M.-R., Chiang Y.-S., Chen P.-Y., Clark A.T. (2016). Stage-Specific Demethylation in Primordial Germ Cells Safeguards against Precocious Differentiation. Dev. Cell.

[99] Okae H., Chiba H., Hiura H., Hamada H., Sato A., Utsunomiya T., Kikuchi H., Yoshida H., Tanaka A., Suyama M. (2014). Genome-Wide Analysis of DNA Methylation Dynamics during Early Human Development. PLOS Genet..

[100] Kobayashi H., Sakurai T., Imai M., Takahashi N., Fukuda A., Yayoi O., Sato S., Nakabayashi K., Hata K., Sotomaru Y. (2012). Contribution of Intragenic DNA Methylation in Mouse Gametic DNA Methylomes to Establish Oocyte-Specific Heritable Marks. PLOS Genet..

[101] Shirane K., Toh H., Kobayashi H., Miura F., Chiba H., Ito T., Kono T., Sasaki H. (2013). Mouse Oocyte Methylomes at Base Resolution Reveal Genome-Wide Accumulation of Non-CpG Methylation and Role of DNA Methyltransferases. PLOS Genet..

[102] Alkhaled Y., Laqqan M., Tierling S., Porto C.L., Amor H., Hammadeh M.E. (2018). Impact of cigarette-smoking on sperm DNA methylation and its effect on sperm parameters. Andrologia.

[103] Laqqan M., Tierling S., Alkhaled Y., Porto C., Solomayer E., Hammadeh M. (2017). Aberrant DNA methylation patterns of human spermatozoa in current smoker males. Reprod. Toxicol..

[104] Jenkins T., James E., Alonso D., Hoidal J., Murphy P., Hotaling J., Cairns B., Carrell D., Aston K. (2017). Cigarette smoking significantly alters sperm DNA methylation patterns. Andrology.

[105] Dura M., Teissandier A., Armand M., Barau J., Lapoujade C., Fouchet P., Bonneville L., Schulz M., Weber M., Baudrin L.G. (2022). DNMT3A-dependent DNA methylation is required for spermatogonial stem cells to commit to spermatogenesis. Nat. Genet..

[106] Uehara R., Yeung W.K.A., Toriyama K., Ohishi H., Kubo N., Toh H., Suetake I., Shirane K., Sasaki H. (2023). The DNMT3A ADD domain is required for efficient de novo DNA methylation and maternal imprinting in mouse oocytes. PLOS Genet..

[107] Greeson K.W., Crow K.M.S., Edenfield R.C., Easley C.A. (2023). Inheritance of paternal lifestyles and exposures through sperm DNA methylation. Nat. Rev. Urol..

[108] Tang W.W., Kobayashi T., Irie N., Dietmann S., Surani M.A. (2016). Specification and epigenetic programming of the human germ line. Nat. Rev. Genet..

[109] Menezo Y.J., Dale B., Elder K. (2019). The negative impact of the environment on methylation/epigenetic marking in gametes and embryos: A plea for action to protect the fertility of future generations. Mol. Reprod. Dev..

[110] Dong H., Wang Y., Zou Z., Chen L., Shen C., Xu S., Zhang J., Zhao F., Ge S., Gao Q. (2017). Abnormal Methylation of Imprinted Genes and Cigarette Smoking: Assessment of Their Association with the Risk of Male Infertility. Reprod. Sci..

[111] Murphy S.K., Itchon-Ramos N., Visco Z., Huang Z., Grenier C., Schrott R., Acharya K., Boudreau M.-H., Price T.M., Raburn D.J. (2018). Cannabinoid exposure and altered DNA methylation in rat and human sperm. Epigenetics.

[112] Schrott R., Murphy S.K., Modliszewski J.L., E King D., Hill B., Itchon-Ramos N., Raburn D., Price T., Levin E.D., Vandrey R. (2021). Refraining from use diminishes cannabis-associated epigenetic changes in human sperm. Environ. Epigenetics.

[113] Schrott R., Rajavel M., Acharya K., Huang Z., Acharya C., Hawkey A., Pippen E., Lyerly H.K., Levin E.D., Murphy S.K. (2020). Sperm DNA methylation altered by THC and nicotine: Vulnerability of neurodevelopmental genes with bivalent chromatin. Sci. Rep..

[114] Vozdova M., Kubickova S., Kopecka V., Pauciullo A., Rubes J. (2024). Impact of air pollution from different sources on sperm DNA methylation. Int. J. Environ. Health Res..

[115] Cheng Y., Tang Q., Lu Y., Li M., Zhou Y., Wu P., Li J., Pan F., Han X., Chen M. (2022). Semen quality and sperm DNA methylation in relation to long-term exposure to air pollution in fertile men: A cross-sectional study. Environ. Pollut..

[116] Schrott R., I Feinberg J., Newschaffer C.J., Hertz-Picciotto I., A Croen L., Fallin M.D., E Volk H., Ladd-Acosta C., Feinberg A.P. (2024). Exposure to air pollution is associated with DNA methylation changes in sperm. Environ. Epigenetics.

[117] Cheng Y., Feng J., Wang J., Zhou Y., Bai S., Tang Q., Li J., Pan F., Xu Q., Lu C. (2023). Alterations in sperm DNA methylation may as a mediator of paternal air pollution exposure and offspring birth outcomes: Insight from a birth cohort study. Environ. Res..

[118] Tian M., Liu L., Zhang J., Huang Q., Shen H. (2019). Positive association of low-level environmental phthalate exposure with sperm motility was mediated by DNA methylation: A pilot study. Chemosphere.

[119] Wu H., Estill M.S., Shershebnev A., Suvorov A., A Krawetz S., Whitcomb B.W., Dinnie H., Rahil T., Sites C.K., Pilsner J.R. (2017). Preconception urinary phthalate concentrations and sperm DNA methylation profiles among men undergoing IVF treatment: A cross-sectional study. Hum. Reprod..

[120] Oluwayiose O.A., Marcho C., Wu H., Houle E., Krawetz S.A., Suvorov A., Mager J., Pilsner J.R. (2021). Paternal preconception phthalate exposure alters sperm methylome and embryonic programming. Environ. Int..

[121] Tadros W., Lipshitz H.D. (2009). The maternal-to-zygotic transition: A play in two acts. Development.

[122] Hertig A.T., Rock J., Adams E.C. (1956). A description of 34 human ova within the first 17 days of development. Am. J. Anat..

[123] Hertig A.T., Rock J., Adams E.C., Mulligan W.J. (1954). On the preimplantation stages of the human ovum: A description of four normal and four abnormal specimens ranging from the second to the fifth day of development. Contrib. Embryol..

[124] Rossant J. (1975). Investigation of the determinative state of the mouse inner cell mass. Development.

[125] Tarkowski A.K., Suwińska A., Czołowska R., Ożdżeński W. (2010). Individual blastomeres of 16- and 32-cell mouse embryos are able to develop into foetuses and mice. Dev. Biol..

[126] Posfai E., Schell J.P., Janiszewski A., Rovic I., Murray A., Bradshaw B., Yamakawa T., Pardon T., El Bakkali M., Talon I. (2021). Evaluating totipotency using criteria of increasing stringency. Nat. Cell Biol..

[127] Guo H., Zhu P., Yan L., Li R., Hu B., Lian Y., Yan J., Ren X., Lin S., Li J. (2014). The DNA methylation landscape of human early embryos. Nature.

[128] Martire S., Banaszynski L.A. (2020). The roles of histone variants in fine-tuning chromatin organization and function. Nat. Rev. Mol. Cell Biol..

[129] Puschendorf M., Terranova R., Boutsma E., Mao X., Isono K.-I., Brykczynska U., Kolb C., Otte A.P., Koseki H., Orkin S.H. (2008). PRC1 and Suv39h specify parental asymmetry at constitutive heterochromatin in early mouse embryos. Nat. Genet..

[130] Li Y., Zhang Z., Chen J., Liu W., Lai W., Liu B., Li X., Liu L., Xu S., Dong Q. (2018). Stella safeguards the oocyte methylome by preventing de novo methylation mediated by DNMT1. Nature.

[131] Andrews S., Krueger C., Mellado-Lopez M., Hemberger M., Dean W., Perez-Garcia V., Hanna C.W. (2023). Mechanisms and function of de novo DNA methylation in placental development reveals an essential role for DNMT3B. Nat. Commun..

[132] Schübeler D. (2015). Function and information content of DNA methylation. Nature.

[133] Mukamel Z., Lifshitz A., Mittnenzweig M., Chomsky E., Schwartzman O., Ben-Kiki O., Zerbib M., Tanay A. (2022). DNA methyltransferases 3A and 3B target specific sequences during mouse gastrulation. Nat. Struct. Mol. Biol..

[134] Moritz L., Hammoud S.S. (2022). The Art of Packaging the Sperm Genome: Molecular and Structural Basis of the Histone-To-Protamine Exchange. Front. Endocrinol..

[135] Yoshida K., Muratani M., Araki H., Miura F., Suzuki T., Dohmae N., Katou Y., Shirahige K., Ito T., Ishii S. (2018). Mapping of histone-binding sites in histone replacement-completed spermatozoa. Nat. Commun..

[136] Inoue A., Zhang Y. (2014). Nucleosome assembly is required for nuclear pore complex assembly in mouse zygotes. Nat. Struct. Mol. Biol..

[137] Schlissel G., Rine J. (2019). The nucleosome core particle remembers its position through DNA replication and RNA transcription. Proc. Natl. Acad. Sci. USA.

[138] Gan H., Serra-Cardona A., Hua X., Zhou H., Labib K., Yu C., Zhang Z. (2018). The Mcm2-Ctf4-Polα Axis Facilitates Parental Histone H3-H4 Transfer to Lagging Strands. Mol. Cell.

[139] Wenger A., Biran A., Alcaraz N., Redó-Riveiro A., Sell A.C., Krautz R., Flury V., Reverón-Gómez N., Solis-Mezarino V., Völker-Albert M. (2023). Symmetric inheritance of parental histones governs epigenome maintenance and embryonic stem cell identity. Nat. Genet..

[140] Li Z., Hua X., Serra-Cardona A., Xu X., Gan S., Zhou H., Yang W.-S., Chen C.-L., Xu R.-M., Zhang Z. (2020). DNA polymerase α interacts with H3-H4 and facilitates the transfer of parental histones to lagging strands. Sci. Adv..

[141] Hackett J.A., Sengupta R., Zylicz J.J., Murakami K., Lee C., Down T.A., Surani M.A. (2013). Germline DNA Demethylation Dynamics and Imprint Erasure Through 5-Hydroxymethylcytosine. Science.

[142] Gruhn W.H., Tang W.W., Dietmann S., Alves-Lopes J.P., Penfold C.A., Wong F.C.K., Ramakrishna N.B., Surani M.A. (2023). Epigenetic resetting in the human germ line entails histone modification remodeling. Sci. Adv..

[143] Karimi M.M., Goyal P., Maksakova I.A., Bilenky M., Leung D., Tang J.X., Shinkai Y., Mager D.L., Jones S., Hirst M. (2011). DNA Methylation and SETDB1/H3K9me3 Regulate Predominantly Distinct Sets of Genes, Retroelements, and Chimeric Transcripts in mESCs. Cell Stem Cell.

[144] Tang W.W., Dietmann S., Irie N., Leitch H.G., Floros V.I., Bradshaw C.R., Hackett J.A., Chinnery P.F., Surani M.A. (2015). A Unique Gene Regulatory Network Resets the Human Germline Epigenome for Development. Cell.

[145] Zhao R., Nakamura T., Fu Y., Lazar Z., Spector D.L. (2011). Gene bookmarking accelerates the kinetics of post-mitotic transcriptional re-activation. Nat. Cell Biol..

[146] Reverón-Gómez N., González-Aguilera C., Stewart-Morgan K.R., Petryk N., Flury V., Graziano S., Johansen J.V., Jakobsen J.S., Alabert C., Groth A. (2018). Accurate Recycling of Parental Histones Reproduces the Histone Modification Landscape during DNA Replication. Mol. Cell.

[147] Petryk N., Dalby M., Wenger A., Stromme C.B., Strandsby A., Andersson R., Groth A. (2018). MCM2 promotes symmetric inheritance of modified histones during DNA replication. Science.

[148] Escobar T.M., Oksuz O., Saldaña-Meyer R., Descostes N., Bonasio R., Reinberg D. (2019). Active and Repressed Chromatin Domains Exhibit Distinct Nucleosome Segregation during DNA Replication. Cell.

[149] Alabert C., Loos C., Voelker-Albert M., Graziano S., Forné I., Reveron-Gomez N., Schuh L., Hasenauer J., Marr C., Imhof A. (2020). Domain Model Explains Propagation Dynamics and Stability of Histone H3K27 and H3K36 Methylation Landscapes. Cell Rep..

[150] Pelham-Webb B., Polyzos A., Wojenski L., Kloetgen A., Li J., Di Giammartino D.C., Sakellaropoulos T., Tsirigos A., Core L., Apostolou E. (2021). H3K27ac bookmarking promotes rapid post-mitotic activation of the pluripotent stem cell program without impacting 3D chromatin reorganization. Mol. Cell.

[151] Aravin A.A., Sachidanandam R., Girard A., Fejes-Toth K., Hannon G.J. (2007). Developmentally Regulated piRNA Clusters Implicate MILI in Transposon Control. Science.

[152] Kuramochi-Miyagawa S., Watanabe T., Gotoh K., Totoki Y., Toyoda A., Ikawa M., Asada N., Kojima K., Yamaguchi Y., Ijiri T.W. (2008). DNA methylation of retrotransposon genes is regulated by Piwi family members MILI and MIWI2 in murine fetal testes. Genes Dev..

[153] Kremsky I., Corces V.G. (2020). Protection from DNA re-methylation by transcription factors in primordial germ cells and pre-implantation embryos can explain trans-generational epigenetic inheritance. Genome Biol..

[154] Festuccia N., Gonzalez I., Owens N., Navarro P. (2017). Mitotic bookmarking in development and stem cells. Development.

[155] Soufi A., Dalton S. (2016). Cycling through developmental decisions: How cell cycle dynamics control pluripotency, differentiation and reprogramming. Development.

[156] Rakyan V.K., E Blewitt M., Druker R., I Preis J., Whitelaw E. (2002). Metastable epialleles in mammals. Trends Genet..

[157] Wolff G.L. (1978). Influence of maternal phenotype on metabolic differentiation of agouti locus mutants in the mouse. Genetics.

[158] Duhl D.M.J., Vrieling H., Miller K.A., Wolff G.L., Barsh G.S. (1994). Neomorphic agouti mutations in obese yellow mice. Nat. Genet..

[159] Belyaev D.K., Ruvinsky A.O., Borodin P.M. (1981). Inheritance of alternative states of the fused gene in mice. J. Hered..

[160] Dickies M.M. (1962). A NEW VIABLE YELLOW MUTATION IN THE HOUSE MOUSE. J. Hered..

[161] Waterland R.A., Jirtle R.L. (2003). Transposable Elements: Targets for Early Nutritional Effects on Epigenetic Gene Regulation. Mol. Cell. Biol..

[162] Fullston T., Teague E.M.C.O., Palmer N.O., DeBlasio M.J., Mitchell M., Corbett M., Print C.G., Owens J.A., Lane M. (2013). Paternal obesity initiates metabolic disturbances in two generations of mice with incomplete penetrance to the F 2 generation and alters the transcriptional profile of testis and sperm microRNA content. FASEB J..

[163] Pereira T.J., Fonseca M.A., Campbell K.E., Moyce B.L., Cole L.K., Hatch G.M., Doucette C.A., Klein J., Aliani M., Dolinsky V.W. (2015). Maternal obesity characterized by gestational diabetes increases the susceptibility of rat offspring to hepatic steatosis via a disrupted liver metabolome. J. Physiol..

[164] Landon M.B., Rice M.M., Varner M.W., Casey B.M., Reddy U.M., Wapner R.J., Rouse D.J., Biggio J.J.R., Thorp J.M., Chien E.K. (2014). Mild Gestational Diabetes Mellitus and Long-Term Child Health. Diabetes Care.

[165] Quilter C.R., Cooper W.N., Cliffe K.M., Skinner B.M., Prentice P.M., Nelson L., Bauer J., Ong K.K., Constância M., Lowe W.L. (2014). Impact on offspring methylation patterns of maternal gestational diabetes mellitus and intrauterine growth restraint suggest common genes and pathways linked to subsequent type 2 diabetes risk. FASEB J..

[166] Alba-Linares J.J., Pérez R.F., Tejedor J.R., Bastante-Rodríguez D., Ponce F., Carbonell N.G., Zafra R.G., Fernández A.F., Fraga M.F., Lurbe E. (2023). Maternal obesity and gestational diabetes reprogram the methylome of offspring beyond birth by inducing epigenetic signatures in metabolic and developmental pathways. Cardiovasc. Diabetol..

[167] Heijmans B.T., Tobi E.W., Stein A.D., Putter H., Blauw G.J., Susser E.S., Slagboom P.E., Lumey L.H. (2008). Persistent epigenetic differences associated with prenatal exposure to famine in humans. Proc. Natl. Acad. Sci. USA.

[168] Yeung E., Biedrzycki R.J., Herrera L.C.G., Issarapu P., Dou J., Marques I.F., Mansuri S.R., Page C.M., Harbs J., Khodasevich D. (2024). Maternal age is related to offspring DNA methylation: A meta-analysis of results from the PACE consortium. Aging Cell.

[169] Cardenas A., Faleschini S., Cortes Hidalgo A., Rifas-Shiman S.L., Baccarelli A.A., DeMeo D.L., Litonjua A.A., Neumann A., Felix J.F., Jaddoe V.W.V. (2019). Prenatal maternal antidepressants, anxiety, and depression and offspring DNA methylation: Epigenome-wide associations at birth and persistence into early childhood. Clin. Epigenet..

[170] Bogdanović O., Smits A.H., Mustienes E.d.l.C., Tena J.J., Ford E., Williams R., Senanayake U., Schultz M.D., Hontelez S., van Kruijsbergen I. (2016). Active DNA demethylation at enhancers during the vertebrate phylotypic period. Nat. Genet..

[171] Greer E.L., Maures T.J., Ucar D., Hauswirth A.G., Mancini E., Lim J.P., Benayoun B.A., Shi Y., Brunet A. (2011). Transgenerational epigenetic inheritance of longevity in Caenorhabditis elegans. Nature.

[172] Beck D., Sadler-Riggleman I., Skinner M.K. (2017). Generational comparisons (F1 versus F3) of vinclozolin induced epigenetic transgenerational inheritance of sperm differential DNA methylation regions (epimutations) using MeDIP-Seq. Environ. Epigenetics.

[173] Macfarlan T.S., Gifford W.D., Driscoll S., Lettieri K., Rowe H.M., Bonanomi D., Firth A., Singer O., Trono D., Pfaff S.L. (2012). Embryonic stem cell potency fluctuates with endogenous retrovirus activity. Nature.

[174] Wu J., Huang B., Chen H., Yin Q., Liu Y., Xiang Y., Zhang B., Liu B., Wang Q., Xia W. (2016). The landscape of accessible chromatin in mammalian preimplantation embryos. Nature.

[175] Fu B., Ma H., Liu D. (2019). Endogenous Retroviruses Function as Gene Expression Regulatory Elements During Mammalian Pre-implantation Embryo Development. Int. J. Mol. Sci..

[176] Xie M., Hong C., Zhang B., Lowdon R.F., Xing X., Li D., Zhou X., Lee H.J., Maire C.L., Ligon K.L. (2013). DNA hypomethylation within specific transposable element families associates with tissue-specific enhancer landscape. Nat. Genet..

[177] Sheikhpour M., Maleki M., Vargoorani M.E., Amiri V. (2021). A review of epigenetic changes in asthma: Methylation and acetylation. Clin. Epigenetics.

[178] Wu Y.-L., Lin Z.-J., Li C.-C., Lin X., Shan S.-K., Guo B., Zheng M.-H., Li F., Yuan L.-Q., Li Z.-H. (2023). Epigenetic regulation in metabolic diseases: Mechanisms and advances in clinical study. Signal Transduct. Target. Ther..

[179] Raghubeer S. (2023). The influence of epigenetics and inflammation on cardiometabolic risks. Semin. Cell Dev. Biol..

[180] Kucher A., Nazarenko M. (2023). Epigenetics of Cardiomyopathy: Histone Modifications and DNA Methylation. Russ. J. Genet..

